# The Institutional Future

**Published:** 1914-11-07

**Authors:** 


### THE INSTITUTIONAL FUTURE.

We published last week an article dealing with "Poor-Law Infirmaries and Union Hospitals," in which an inquiry was suggested with a view to placing on record the uses to which each type of institution is being put, so that the Government might be in a position really to know what in-patient accommodation might be made available, in existing institutions, to deal with the increased demand for beds which the Insurance Act continues to occasion. In case it might be thought that the present time is not one best suited to inquiries of this character, and that each Government department is too much occupied with the stress of affairs that is everywhere being felt, we propose this week to show that the request for this inquiry was not casually timed, but deliberately chosen in view of a reasonable forecast of the future.

One thing only is tolerably certain about the European situation, and that is that the titanic struggle in which the nations are engaged will not be quickly over, or, which comes to the same thing, that neither side will lay down their arms until the distress in their own borders has become so acute as to be intolerable. This means in practice that the institutions in each country will be tested as they have not been tested for generations, and the reaction on every type of national institution—constitutional, political, and social—will be correspondingly far-reaching. The War is throwing not merely our armies but our civilisations into the melting-pot, and we can be sure only that changes which might not have come about in the next fifty years, had peace been maintained, are likely within five years to be pressing themselves upon us. There is nothing necessarily alarmist in this statement of fact; but it is well to keep in view what has already begun to happen, even in the relatively circumscribed field of hospital work.

The Insurance Act created even more problems than it attempted to solve, and the first of its definite achievements was to place on record an amount of national sickness which, till its means for recording health statistics came into being, was quite unsuspected and unrecognised. In vain do the approved societies clamour for medical referees; in vain is there an outcry against excessive prescribing; in vain is malingering supposed to be the cause of impending insolvency. The truth is that far more sickness exists than had been calculated for. The consequent demand for in-patient treatment, with its revelation of the insufficiency of hospital beds, has led to the beginning of co-operation between public and voluntary institutions, the need for which was pointed out in The Hospital, largely to deaf ears, long before facts drove it home unanswerably. Here, then, we find already the symptoms, nay, the process, of change. This process the War can serve only to hasten.

Already the numbers of wounded continuing inevitably to arrive in this country are making us realise that our existing means of accommodation are not unlimited; and one type of institution after another, including the private nursing home, is being utilised. What effects will this have on our hospital system? Plainly, its first result will be that tendency to break down arbitrary distinctions which is the essence of co-operation; the welding together of all institutions engaged in allied work under a common scheme. The Poor Law and the public authorities, as well as the voluntary hospitals, are all playing their part, and an increasingly common part in the national emergency. It is not too much, then, to state that wise men will not be content to drift with the tide, but rather will determine to direct its force into considered channels; and that is why no time is likely to occur more fruitful than the present for an inquiry on the part of the Local Government Board into the relative uses of Poor-Law infirmaries and Union hospitals. The State hospital is emerging by force of circumstance. Now is the time for that co-operative scheme, securing more firmly than ever the preservation of the voluntary principle, extended by graduated pay wards, and public institutions, properly co-ordinated, for which we have pleaded so often in the past.

